# Editorial: The psychology of love

**DOI:** 10.3389/fpsyg.2024.1518730

**Published:** 2024-12-11

**Authors:** Thiago de Almeida, David L. Rodrigues

**Affiliations:** ^1^Department of Religious Sciences, Pontifícia Universidade Católica (PUC-Goiás), Goiás, Brazil; ^2^Iscte-Instituto Universitário de Lisboa, CIS-Iscte, Lisbon, Portugal

**Keywords:** psychology of love, positive psychology, romantic love, love, romantic relationships dyadic approach

Understanding romantic love has been among the central Research Topics in psychology since the 1950s, given its benefits for individual health and wellbeing. In one of the earliest studies in this field, Rubin (1970) developed a scale to assess romantic love and distinguish it from liking and argued that romantic love includes three main components—attachment, caring, and intimacy (Rubin, 1973). This seminal work was crucial to inform the development of theoretical framework widely used (for a review, see Sternberg, 2018). Despite the many difficulties in objectively assessing love (Hendrick and Hendrick, 2019), researchers continue to examine how people experience love, its personal, relational, and contextual correlates, and its implications for functioning. For example, some researchers recently proposed new theories to explain the meaning and experience of love (e.g., Tobore, 2020), whereas others tested the generalizability of established scales (e.g., Triangular Love Scale; Sorokowski et al., 2021). Likewise, some studies have shown that emotional support and involvement in stable romantic relationships can reduce stress reactivity and have positive effects on health (e.g., Coan et al., 2006). Other studies have shown that romantic relationships perceived as valuable by both partners are associated with significant improvements in mental health, including decreased depression, anxiety, and loneliness (e.g., Proulx et al., 2007). Some of these associations have been replicated in longitudinal studies. For example, individuals in stable, high-quality romantic relationships tend to live longer and exhibit lower mortality rates, compared to those who are single or divorced (Holt-Lunstad et al., 2010; Bouchard et al., 2023; Sheng et al., 2023).

In particular, the Psychology of Love has stood out as an interdisciplinary field that investigates multiple dynamics involved in romantic relationships. In recent years, academic interest has evolved considerably, incorporating new perspectives that go beyond traditional approaches to interpersonal attraction, manifestation of affection, and related sexual practices (e.g., sexually diverse individuals; relational diversity; Da Silva et al., 2005; Almeida, 2008, 2010, 2012; Almeida and Lourenço, 2011; Almeida et al., 2008; Sousa et al., 2009; Antunes et al., 2010; Franklin et al., 2015; Lima and Almeida, 2016; Hatakeyama et al., 2017; Almeida and Dourado, 2018; Almeida and Lomônaco, 2018). As Western societies become more complex and human interactions occur in multiple contexts, a deeper and broader understanding of love and its implications becomes necessary.

This Research Topic includes studies exploring different aspects related to the experience of love in samples from around the world:

*Individuals' traits and beliefs*: Pirrone et al. examined how attachment styles shaped emotional regulation during conflicts in romantic relationships in Belgian individuals. The authors found that negative disengaging emotions (e.g., anger and irritation) were associated with autonomy frustration, whereas negative engaging emotions (e.g., sadness, hurt, and disappointment) were linked to relatedness frustration. The study also revealed that individuals' relationship beliefs moderated the intensity of these emotions, especially regarding relatedness frustration. In another study, Yilmaz et al. showed that traumatic experiences (e.g., parental divorce) negatively shaped trust in future relationships among Turkish university students. This erosion of trust was linked to attachment styles, such that individuals who reported more anxious or avoidant attachment styles experienced more difficulties in maintaining trusting relationships. Moreover, Tartakovsky identified core romantic motivations, such as love and care, family, and status, that reflect individual values and shape relational dynamics in a sample of young individuals in Israel. According to the author, the alignment between these motivations and personal values plays a critical role in the formation and maintenance of romantic relationships.

*Expectations and preferences*: Thompson et al. investigated the link between idealized first romantic kiss beliefs and romantic love among adults in the United States. Their results indicated that greater endorsement of idealized first kiss beliefs was correlated with higher levels of romantic love. Romantic attachment moderated this link, highlighting the importance of these beliefs for individuals with higher attachment insecurity. In a revision of the literature, Besika examined the link between happiness and meaning in life and proposed that happiness (as the experience of positive emotions) can serve as an indicator of wellbeing, whereas meaning (as ongoing cognitive processes) can help maintain wellbeing. In their study, Liu and Zhang examined the role of similarity in partner selection among Chinese individuals who were single. The authors showed that individuals often prefer partners with similar personality traits. This preference, however, may not be universal and can be shaped by individual factors, like loneliness or perceived self-worth.

*Infidelity and jealousy*: Fernandez et al. found that love was correlated with jealousy among individuals in Chile jealousy and argued that both experiences serve adaptive functions. For the authors, jealousy plays a role in preserving romantic bonds when expressed in a healthy manner. In their study, Kato and Okubo examined reactions to infidelity among individuals in Japan and found that both women and men in committed relationships reported greater distress over sexual infidelity. Their findings challenge traditional evolutionary psychology views, indicating that relationship status can be more determinant of infidelity reactions than inherent gender differences.

*Contextual determinants*: Guzmán-González et al. explored how internalized negative feelings over one's sexual identity shaped emotional intimacy among gay male couples in Chile. Internalized homonegativity was found to exacerbate the negative role of attachment insecurity, making it difficult for individuals to achieve emotional closeness. Examining broader environmental factors, Cheng et al. provided new perspectives on how events, such as the COVID-19 pandemic, might have determined the dynamics of romantic relationships among Chinese college students. Their research showed that societal crises can have downstream consequences for subjective wellbeing and relationship perceptions, underlining the importance of considering the sociocultural context in the study of love.

In conclusion, the study of love is vast and ever evolving, encompassing not only the positive aspects of the romantic experience, but also its challenges, such as jealousy, conflict, and insecurity. Interdisciplinary research offers valuable insights to psychologists and theorists, facilitating more comprehensive views, informing the development of more effective interventions, and promoting healthy and resilient relationships. Enjoy the reading!
